# Multidimensional Optimization of *Saccharomyces cerevisiae* for Carotenoid Overproduction

**DOI:** 10.34133/bdr.0026

**Published:** 2024-01-10

**Authors:** Jian Fan, Yang Zhang, Wenhao Li, Zhizhen Li, Danli Zhang, Qiwen Mo, Mingfeng Cao, Jifeng Yuan

**Affiliations:** ^1^ State Key Laboratory of Cellular Stress Biology, School of Life Sciences, Faculty of Medicine and Life Sciences, Xiamen University, Fujian 361102, China.; ^2^ Shenzhen Research Institute of Xiamen University, Shenzhen 518057, China.; ^3^ College of Chemistry and Chemical Engineering, Xiamen University, Fujian 361005, China.; ^4^ Key Laboratory for Synthetic Biotechnology of Xiamen City, Xiamen University, Fujian 361005, China.

## Abstract

Microbial synthesis of carotenoids is a highly desirable alternative to plant extraction and chemical synthesis. In this study, we investigated multidimensional strategies to improve the carotenoid synthesis in the industrial workhorse of *Saccharomyces cerevisiae*. First, we rewired the yeast central metabolism by optimizing non-oxidative glycolysis pathway for an improved acetyl-CoA supply. Second, we restricted the consumption of farnesyl pyrophosphate (FPP) by the down-regulation of squalene synthase using the PEST degron. Third, we further explored the human lipid binding/transfer protein saposin B (hSapB)-mediated metabolic sink for an enhanced storage of lipophilic carotenoids. Last, the copper-induced GAL expression system was engineered to function in the yeast–peptone–dextrose medium for an increased biomass accumulation. By combining the abovementioned strategies, the final engineered yeast produced 166.79 ± 10.43 mg/l β-carotene in shake flasks, which was nearly 5-fold improvement of the parental carotenoid-producing strain. Together, we envision that multidimensional strategies reported here might be applicable to other hosts for the future industrial development of carotenoid synthesis from renewable feedstocks.

## Introduction

Carotenoids are widely occurring isoprenoids in nature, mainly found in higher plants, algae, and microorganisms [1]. Due to their antioxidant properties, carotenoids have been extensively used in medicine, healthcare, food, and cosmetics industries [2]. Carotenoids belong to the class of tetraterpenoids consisting of a basic skeleton of C_40_H_56_, and they are mainly divided into two categories: Carotenes include lycopene, α-carotene, β-carotene, and γ-carotene; xanthophylls include zeaxanthin, lutein, and astaxanthin. Among them, both β-carotene and lycopene have good abilities to quench singlet oxygen and capture free radicals, which are used for the treatment of cardiovascular diseases and prostate cancer [3]. Especially, astaxanthin as one of the strongest antioxidants has the potential for anti-aging effect, sun-blocking effect, and anti-inflammatory effect when administered with aspirin [4]. In addition, carotenoids can also be converted to retinoids such as vitamin A, which are applicable in pharmaceuticals, foods, nutraceuticals, cosmetics, and animal feed additives [5].

β-Carotene and astaxanthin are two representatives of commercially available carotenoids on the market. More than 90% of β-carotene and astaxanthin are produced by chemical synthesis, which is developed by Roche and BASF Corporation [6]. Because of the increasing standard for living and consumption, there is an arising demand to replace chemically synthesized nutraceuticals with natural compounds. Although carotenoids can be extracted from plants and algae [6], they are still limited by time and space, and the high cost of extraction process. With the continuous development of synthetic biology and metabolic engineering, microbial synthesis of natural carotenoids is rapidly expanding in recent years [7,8]. It was reported that a variety of microorganisms such as *Escherichia coli*, *Saccharomyces cerevisiae*, and *Yarrowia lipolytica* have been established for carotenoid synthesis by introducing the heterologous carotenogenic genes [9,10]. Several useful strategies have been accomplished in different microorganisms to improve the carotenoid synthesis. For instance, the combination of the hybrid mevalonate (MVA) pathway and the optimized 1-deoxy-d-xylulose 5-phosphate (DXP) pathway with the heterologous geranyl diphosphate synthase resulted in a 113-fold increase of β-carotene titer in *E. coli* [11]. An alternative cytoplasmic acetyl-CoA (coenzyme A) pathway was introduced in *Y. lipolytica* to increase the supply of acetyl-CoA, resulting in a 32% enhanced β-ionone titer [12]. To increase the supply of NADPH (reduced form of nicotinamide adenine dinucleotide phosphate) for the isoprenoid biosynthesis, the individual overexpression of glucose-6-phosphate dehydrogenase and NADH [reduced form of nicotinamide adenine dinucleotide (oxidized form)] kinase in a carotenoid-producing strain of *S. cerevisiae* reached 59.9% and 81.4% increases of lycopene and β-carotene titers [13]. Moreover, cell morphology engineering and inner- and outer-membrane vesicle formation were applied in *E. coli* to significantly enhance three hydrophobic compounds belonging to carotenoids [14]. Although many attempts have been made in improving the microbial production of carotenoids, the carotenoid productivity by microbial fermentation is suboptimal for meeting the needs of industrial production.

**Fig. 1.**
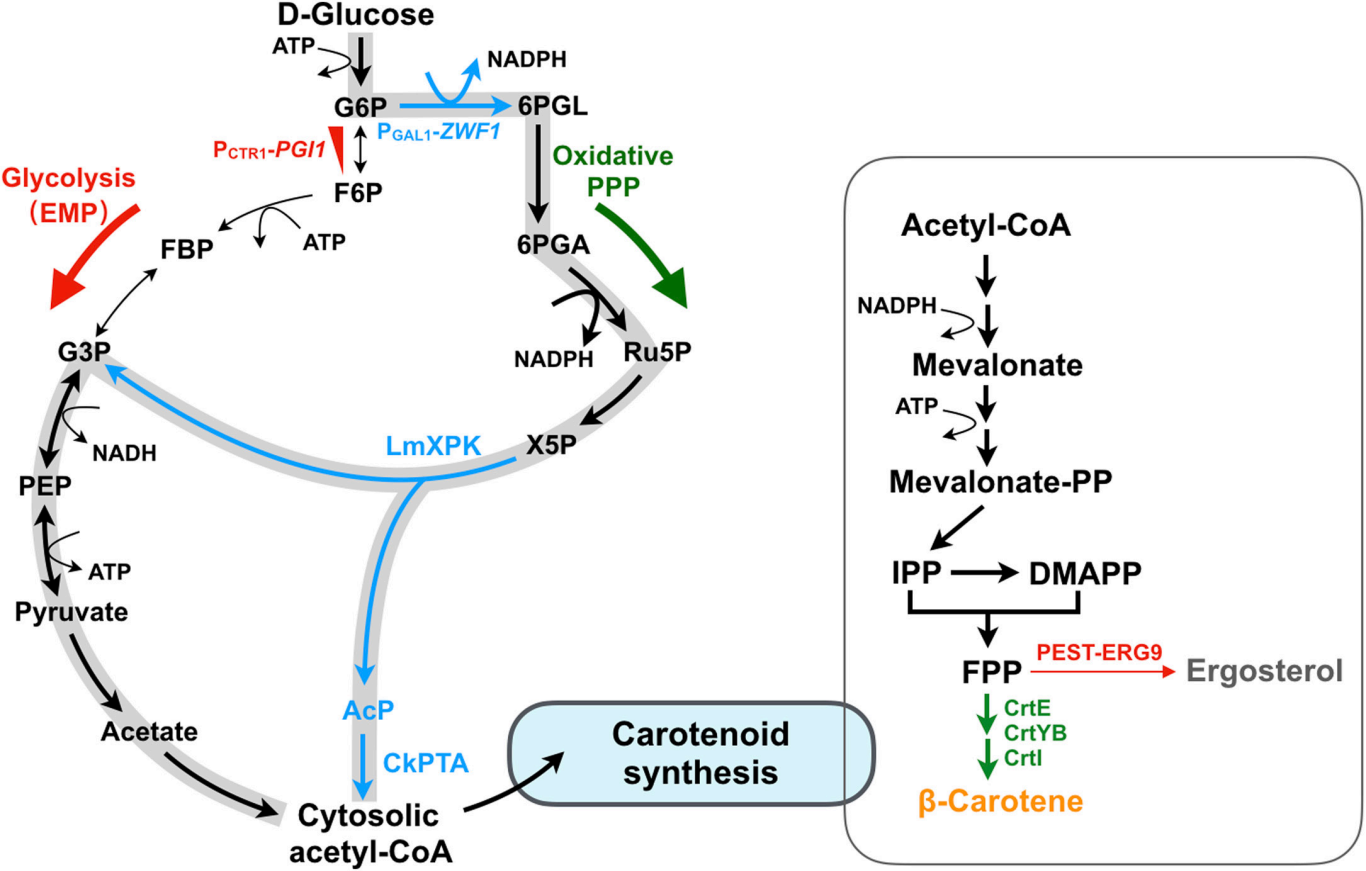
Schematic diagram of β-carotene synthesis in *S. cerevisiae*. β-Carotene can be synthesized from acetyl-CoA via the MVA pathway together with the heterologous expression of CrtE, CrtYB, and CrtI from *X. dendrorhous*. P_CTR1_-*PGI1* indicates phosphoglucose isomerase under the control of copper-repressible CTR1 promoter. EMP, Embden–Meyerhoff–Parnas; LmXPK, xylulose-5-phosphate-specific phosphoketolase from *L. mesenteroides*; CkPTA, phosphotransacetylase from *C. kluyveri*; G6P, glucose-6-phosphate; 6PGL, 6-phosphogluconolactone; 6PGA, 6-phosphogluconic acid; Ru5P, ribulose 5-phosphate; X5P, xylulose-5-phosphate; AcP, acetyl-phosphate; F6P, fructose-6-phosphate; FBP, fructose-1,6-bisphosphatase; PEP, phosphoenolpyruvate; G3P, glyceraldehyde 3-phosphate.

In this study, we aimed to investigate multidimensional strategies based on the model microorganism of *S. cerevisiae* to improve the microbial synthesis of carotenoid (Fig. 1). First, we rewired the central metabolism by introducing phosphoketolase (PK) and phosphotransacetylase (PTA) pathway together with restricting the glycolytic pathway to improve the acetyl-CoA supply. Second, the down-regulation of the squalene synthase (encoded by *ERG9*) using the PEST degron was employed to restrict the consumption of FPP to ergosterol pathway. Third, the human lipid binding/transfer protein saposin B (hSapB) was introduced for an enhanced storage of lipophilic carotenoids. In the end, the copper-induced GAL expression system was engineered to function in the rich yeast–peptone–dextrose (YPD) medium for a better accumulation of biomass. By combining different engineering strategies, the final engineered yeast produced ~5-fold improvement of β-carotene over that of the parental carotenoid-producing strain, reaching 166.79 mg/l β-carotene in YPD under shake-flask conditions.

## Materials and Methods

### Strains and reagents

*E. coli* TOP10 was used for the routine construction of plasmids, and *E. coli* strains transformed with plasmids were cultivated at 37 °C in Luria–Bertani broth with 100 μg/ml ampicillin. *S. cerevisiae* JS-BE (a derivative from BY4741 for carotenoid synthesis) [5] was used as the starting strain for the subsequent generation of carotenoid-producing yeast strain. *S. cerevisiae* JS-BE was cultivated in rich YPD medium, and the engineered strains with auxotrophic selection markers were maintained in the yeast–nitrogen–base dextrose (YNBD) medium with dropouts. All restriction enzymes, Phusion High-Fidelity DNA polymerase, and T4 ligase were purchased from New England Biolabs (Beverly, MA, USA). The commercial kits for gel extraction and plasmid purification were both obtained from BioFlex (Shanghai, China).

### Plasmid construction and yeast transformation

All the oligonucleotides used in this study are listed in Table S1. The in-house designed plasmid pRS425-GGA [15] was employed for cloning the expression cassette using *Bsa*I-mediated golden-gate assembly method. The xylulose-5-phosphate-specific PK from *Leuconostoc mesenteroides* (LmXPK) and PTA from *Clostridium kluyveri* (CkPTA) were codon-optimized and synthesized by GenScript (Nanjing, Jiangsu, China), and the resulting plasmid of pRS425-LmXPK/CkPTA was used as the template for the polymerase chain reaction (PCR) amplification of genome integration cassette. We used CRISPR/Cas9 approach for markerless engineering of the yeast chromosomes, and standard electroporation with a minimal modification was used to transform *S. cerevisiae*. All genome-engineered yeast strains were subjected to diagnostic PCR to confirm the correct events. The guide RNA (gRNA)-expressing plasmid was removed via counter-selection using 5-fluorootic acid (5-FOA; 1 g/l), and the Cas9-expressing plasmid was eliminated under nonselective conditions. Overall, six rounds of genetic modifications were implemented to yield ∆gpp1:LmXPK/CkPTA, ∆gal7/10/1:Zwf1, ∆PGI1P:P_CTR1_, PEST-ERG9N, ∆oye2:hSpaB, and PEST-Gal80N. All plasmids and strains used in the present study are listed in Table S2.

### Carotenoid productions in shake flasks

For small-scale carotenoid production, experiments were carried out using 100 ml of shake flasks. In brief, fresh overnight yeast cultures were inoculated into 20 ml of YPD or YNBD media containing 2% (w/v) glucose with appropriate dropouts to an initial OD_600_ (optical density at 600 nm) of 0.1. Copper sulfate (20 μM) was supplemented to induce the genes under the control of copper-inducible GAL system. After 72-h cultivation, 100 μl of fermentation broth was taken for the measurement of OD_600_ with a microplate reader (Synergy H1, Biotek, USA).

The intracellular contents of carotenoids were extracted from the yeast cell pellets using acetone. In specific, 1 ml of cell culture was sampled and centrifuged to remove the supernatant. The harvested cells were then resuspended with 1 ml of acetone in the screwed cap tubes and crushed by a bead ruptor (OMNI, USA). After centrifugation, the cell debris was discarded and the supernatants containing the carotenoids were aliquoted in new brown tubes to protect carotenoids from light-induced degradation. The acetone extracts were analyzed with high-performance liquid chromatography (HPLC; model LC-20A, Shimadzu) equipped with a C18 column (250 mm × 4.6 mm, 5 μm). The mobile phases were 50:50 methanol and acetonitrile. During the HPLC analysis, the flow rate was maintained at 1.0 ml/min and the column temperature was set at 40 °C. The detection wavelength for carotenoids used in this study was 450 nm. All authentic lycopene and β-carotene standards were dissolved in methanol for the subsequent plotting of the standard curve.

## Results and Discussion

### Metabolic reconfiguration for an improved acetyl-CoA supply

As shown in Fig. 1, the universal precursors for carotenoids are dimethylallyl diphosphate (DMAPP) and isopentenyl diphosphate (IPP), which are generated from acetyl-CoA as the starting substrate though the MVA pathway in yeast. The main source of cytosolic acetyl-CoA is produced from pyruvate catalyzed by pyruvate dehydrogenase, acetaldehyde dehydrogenase, and acetyl-CoA synthetase in budding yeast [16]. Besides, adenosine triphosphate (ATP)-citrate lyase (ACL)-mediated acetyl-CoA generation approach was demonstrated to be effective to improve fatty acid-derived chemicals in budding yeast [17]. Recently, an artificial non-oxidative glycolysis (NOG) pathway with PK and PTA to achieve a 100% carbon yield was first established in *E. coli* [18]. Subsequently, the NOG pathway was explored in budding yeast to rewrite the central carbon metabolism for industrial isoprenoid production [19]. In particular, the combination of PK and PTA enabled the biosynthesis of cytosolic acetyl-CoA in *S. cerevisiae*, which reduced ATP requirement and loss of carbon to CO_2_-emitting reactions. In addition, the NOG pathway can replace the native glycolytic pathway [Embden–Meyerhoff–Parnas (EMP)] in *E. coli* for sugar catabolism [20].

**Fig. 2.**
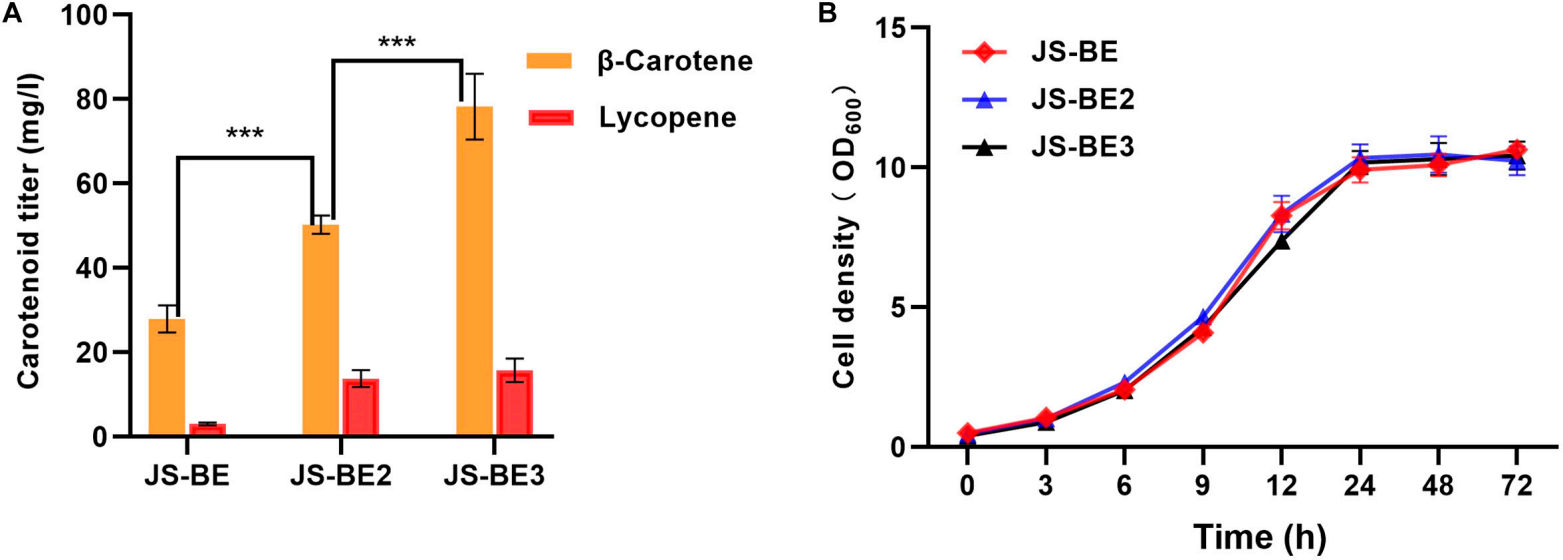
The effect of rewiring the central metabolism on carotenoid synthesis. (A) Carotenoid contents of engineered yeasts in YNBD media at 72 h. Strain JS-BE is the parental carotenoid-producing strain; strain JS-BE2 is a JS-BE derivative with an integration of Δgpp1:LmXPK/CkPTA and an overexpression of Zwf1; strain JS-BE3 is a JS-BE2 derivative with P_CTR1_-*PGI1*. (B) Growth curves of the engineered strains in YNBD. *P* values were obtained from two-tailed unpaired Student’s *t* test. ***P < 0.01. Error bars indicate the SDs of three independent experiments.

Here, we optimized the PK/PTA pathway (Fig. 1) by strengthening the pentose phosphate (PPP) pathway and restricting EMP pathway to improve the supply of acetyl-CoA for carotenoid synthesis in *S. cerevisiae*. The β-carotene-producing *S. cerevisiae* JS-BE was used as the base strain for the subsequent study, which contained an improved β-carotene synthetic pathway under the control of copper-inducible GAL system [5]. To assemble the PK/PTA pathway in JS-BE, we chose to overexpress xylulose-5-phosphate-specific PK from *L. mesenteroides* (LmXPK) [19] and PTA from *C. kluyveri* (CkPTA) [19] under the control of GAL1/10 promotor, and the LmXPK/CkPTA cassette was integrated at the *gpp1* locus to minimize acetate formation in a similar way to previously reported [19]. Moreover, we also engineered the PPP pathway by overexpressing the key rate-limiting step of Zwf1 (glucose-6-phosphate 1-dehydrogenase) to improve the NADPH supply and to enhance the flux toward the production of xylulose-5-phosphate. As shown in Fig. 2A, after 72-h cultivation in YNBD media with 20 μM copper inducer, strain JS-BE2 (JS-BE derivative with ∆gpp1:LmXPK/CkPTA and an overexpression of Zwf1) produced 50.29 mg/l β-carotene and 15.75 mg/l lycopene, which were improved by 80% and 353% when compared with those of JS-BE, respectively. The accumulation of lycopene as an intermediate metabolite suggested the insufficient activity of lycopene cyclase from the bifunctional CrtYB. To limit the EMP pathway for further redirecting the metabolic flux to the NOG pathway, we used the copper-repressible promotor P_CTR1_ to replace the native promoter of phosphoglucose isomerase (encoded by *PGI1*). The resulting strain JS-BE3 (a derivative of JS-BE2 with P_CTR1_-*PGI1*) produced 105.94 mg/l of β-carotene, which represents a nearly 56% improvement compared to JS-BE2. However, there was no significant difference in lycopene contents between strain JS-BE3 and JS-BE2. According to a recent study, knockout of *PFK1/2* encoding phosphofructokinase to abolish fructose-1,6-bisphosphatase synthesis resulted in a nonviable yeast [21]. Surprisingly, the restriction of the EMP pathway by P_CTR1_-*PGI1* did not negatively impact the cell growth of strain JS-BE3 when compared to JS-BE and JS-BE2, indicating that the NOG with strengthened PPP pathway provided sufficient energy molecules and acetyl-CoA to support the cell growth. When compared to the previous design of NOG pathway in budding yeast for isoprenoid production [19], we demonstrated that it is not a must to rely on the transketolase and transaldolase cycle for regenerating xylulose-5-phosphate from fructose-6-phosphate. By restricting phosphoglucose isomerase and strengthening PPP pathway, we optimized the NOG pathway to facilitate acetyl-CoA supply with an enhanced carotenoid synthesis in *S. cerevisiae*. Since there are a variety of XPK and PTA from different sources that showed substantial differences in enzymatic activities [22], future examination of other XPK and PTA candidates might be able to further improve the acetyl-CoA supply.

### A PEST degron to ERG9 for reduced flux toward ergosterol synthesis

In *S. cerevisiae*, the squalene synthase ERG9 represents the key branching point for ergosterol biosynthesis, which competes with carotenoid synthesis for the FPP (an important intermediate) [23]. However, direct disruption of ergosterol synthesis by deleting *ERG9* is undesirable due to the essentiality of ergosterol for cell survival. In order to produce high-level non-native isoprenoids, it is necessary to redirect the metabolic flux from ergosterol synthesis to the heterologous metabolic reactions by down-regulation of *ERG9*. A series of promoters including methionine-repressible MET3 promoter [24], copper-repressible CTR3 promoter [25], glucose-regulated HXT1 promoter [26], and ergosterol-responsive promoters [27] were employed to down-regulate *ERG9* expression.

**Fig. 3.**
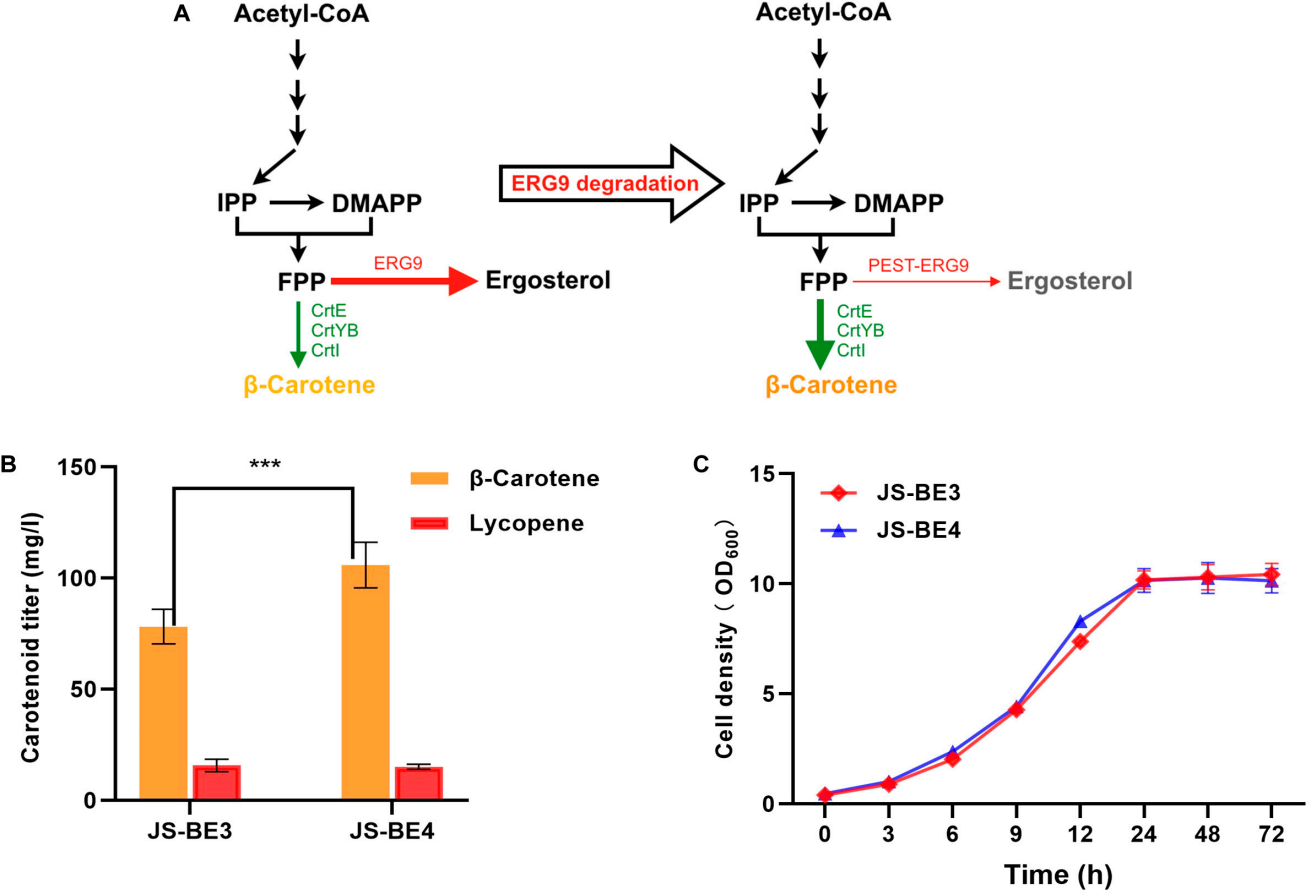
The effect of PEST-ERG9N on carotenoid synthesis. (A) Schematic diagram of PEST-mediated ERG9 degradation to restrict ergosterol synthesis. (B) Carotenoid contents of engineered yeasts in YNBD media at 72 h. Strain JS-BE4 is a JS-BE3 derivative with PEST-ERG9N. (C) Growth curves of JS-BE3 and JS-BE4 in YNBD media. *P* values were obtained from two-tailed unpaired Student’s *t* test. ***P < 0.01. Error bars indicate the SDs of three independent experiments.

There are also other available strategies to restrict the ergosterol biosynthesis such as accelerated ERG9 degradation [28]. For instance, the auxin-inducible degron (AID) system-mediated degradation of ERG9 was implemented in *S. cerevisiae* for an improved isoprenoid production [28]. Based on the literature, the PEST sequence comprising amino acids of L-proline (P), L-glutamic acid (E), L-serine (S), and L-threonine (T) is widely distributed in mammalian cells, plants, and yeast [29], and it is one of the most common motifs for protein degradation through the 26S proteasome pathway [30]. As ERG9 is a membrane-associated protein located in endoplasmic reticulum (ER), we decided to fuse the PEST sequence at the N terminus of ERG9 in strain JS-BE3 (Fig. 3A) to reduce the stability of ERG9 before entering ER. As shown in Fig. 3B, the β-carotene titer of strain JS-BE4 with PEST-ERG9N was increased by 35% in comparison to that of JS-BE3, reaching 105.94 mg/l β-carotene at 72 h. The lycopene level in strain JS-BE4 remained almost the same as that of JS-BE3, suggesting that the reinforced metabolic flux did not further perturb the substrate inhibition to CrtYB. Furthermore, the suppression of ergosterol biosynthesis by PEST-ERG9N had no adverse effect on cell growth (Fig. 3C). These results reflected that reducing the ERG9 stability by the PEST sequence can divert more metabolic flux from ergosterol to carotenogenic reactions. Recently, the replacement of the native ERG9 promoter with an oleic acid-repressible promoter (P_IZH1_) resulted in a 31.7% increase of β-carotene titer in *S. cerevisiae* [23], indicating that the destabilization of ERG9 mediated by the PEST tag had a comparable effect when compared to the conventional promoter engineering. In the future, the PEST-tagged ERG9 might be combined with the previously established ergosterol-responsive promoters [27] to construct more accurate and effective *ERG9* repression systems.

### The effect of an additional metabolic sink for carotenoid storage

Carotenoids such as lycopene and β-carotene are hydrophobic compounds that can only be stored in the lipid droplets or membranes, and the production of carotenoids is typically limited by the storage space inside the cell. One typical solution is transporter-mediated secretion that enables products to be pumped outside the cell, but the efficiencies of transporter-mediated carotenoid secretion are still unsatisfactory for lycopene and β-carotene [31,32]. For instance, Bu *et al*. [33] constructed a β-carotene-producing strain of *S. cerevisiae* by overexpressing the ABC transporter of SNQ2, increasing ATP supply and improving the membrane fluidity for the secreted production of β-carotene, to acquire a titer of 149.8 mg/l from glucose in shake flasks. Another useful solution is to enlarge the intracellular storage capacity by reprogramming the cellular membrane morphology, but unwanted cellular responses are accompanied [34]. There are also other solutions to manipulate the storage space for enhanced carotenoid biosynthesis. For instance, the improved lipid oil-triacylglycerol metabolism was reported to increase the lycopene accumulation by 25% in *S. cerevisiae* by overexpressing a fatty acid desaturase (encoded by *OLE1*) and deleting *FLD1* to regulate the lipid droplet size [35]. Poly-3-hydroxybutyrate (PHB), a hydrophobic biopolymer, was also explored as the intracellular storage vessel to encapsulate lycopene [36].

**Fig. 4.**
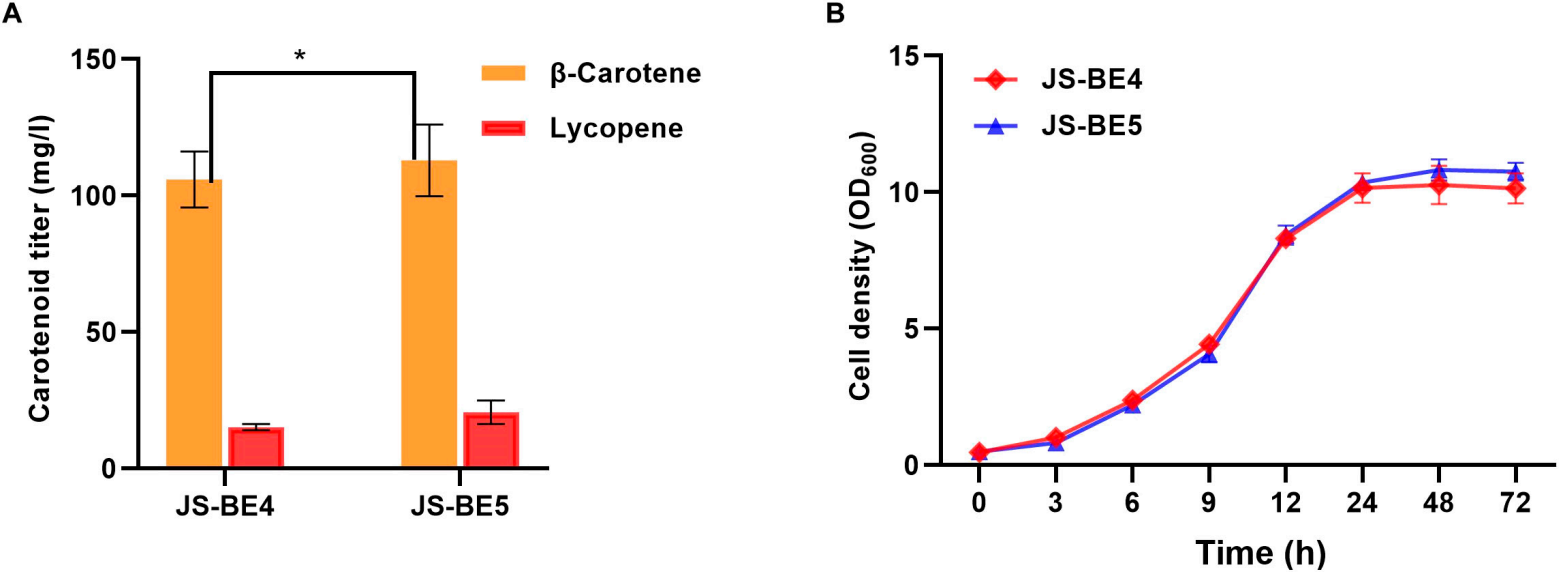
The effect of an additional metabolic sink for storing the intracellular carotenoids. (A) Carotenoid contents of engineered yeasts in YNBD media at 72 h. JS-BE5 is a JS-BE4 derivative with an integration of Δoye2:hSapB. (B) Growth curves of JS-BE4 and JS-BE5 in YNBD. *P* values were obtained from two-tailed unpaired Student’s *t* test. *P < 0.05. Error bars indicate the SDs of three independent experiments.

Previously, our group has demonstrated that hSapB-mediated metabolic sink was capable of increasing coenzyme Q production by enhancing the storage space for lipophilic compounds [37]. More recently, coupling hSapB with secretion signal peptides in *S. cerevisiae* could transport β-carotene and squalene into extracellular medium, and a single SapB protein was reported to bind and transport multiple lipophilic compounds [38]. Here, we constructed strain JS-BE5 with an overexpression of hSapB to improve carotenoid synthesis by introducing an additional metabolic sink. As shown in Fig. 4, the engineered strain JS-BE5 with hSapB overexpression did not show any noticeable change of growth profile, and it produced 112.92 mg/l β-carotene and 20.58 mg/l lycopene. Although the titer of β-carotene in strain JS-BE5 only marginally increased, the lycopene level was improved by 35% when compared with that of strain JS-BE4. It was reported that the triacylglycerol metabolism of lipid bodies was observably improved by adding 565 mg/l oleic acid, leading to a 36.4% increase of β-carotene content in the engineered *S. cerevisiae* [23], and supplementation of 60 mg/l oleic acid and palmitoleic acid resulted in 83.7% and 130.2% increases of β-carotene content in the recombinant *S. cerevisiae*, respectively [39]. Compared to engineering the intracellular lipid formation for carotenoid storage [23,35,39], endogenous production of lipid-binding protein hSapB is relatively simple to manipulate. According to the literature, the engineered yeast cells with carotenogenic genes from *Xanthophyllomyces dendrorhous* predominantly accumulate phytoene, an intermediate substrate of CrtI (phytoene desaturase) [40]. Therefore, the poor improvement of β-carotene synthesis might be explained by the preloading of hSapB with other lipophilic compounds such as phytoene and lycopene; however, future experiments are required to confirm the abundance of phytoene. Since a variant of CarRP^Y27R^ from *Mucor circinelloides* completely relieved the substrate inhibition of lycopene in *Y. lipolytica* [41], it might be possible to employ CarRP^Y27R^ to favor the accumulation of β-carotene over lycopene. More importantly, the accumulation of phytoene intermediate [40] should be addressed by mining more effective CrtI before the metabolic sink system can be implemented to give a substantial improvement on β-carotene production. Considering that an elevated expression level of hSapB could facilitate more secreted carotenoid production [38], it is worth to further investigate the effect of hSapB dosage on carotenoid productions.

### Engineering the copper-induced GAL system to function in YPD medium

**Fig. 5.**
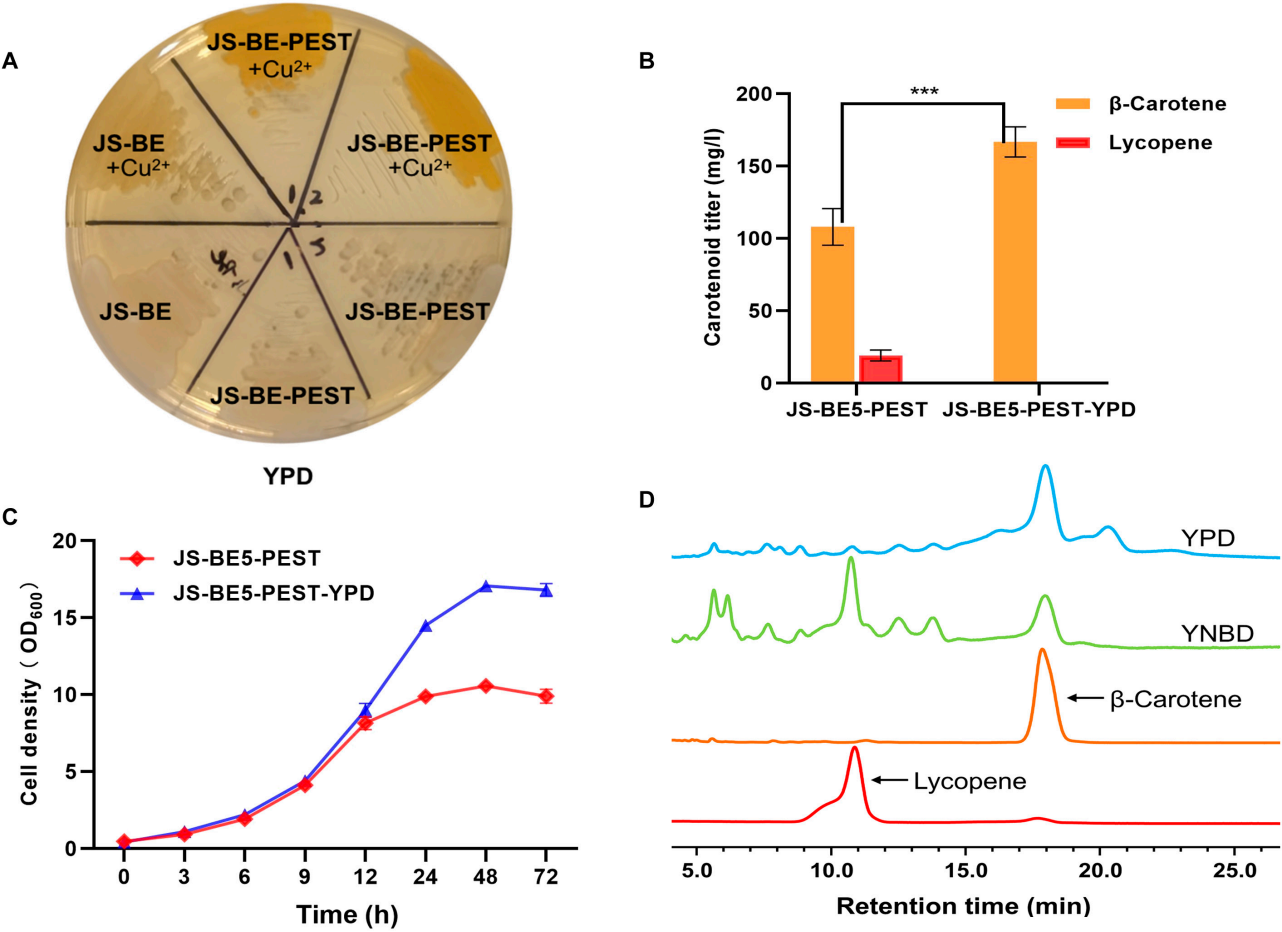
Engineering the GAL system to function in YPD medium for an improved carotenoid storage. (A) PEST fusion to Gal80 for copper-induced GAL expression in YPD media. Copper sulfate (20 μM) was used to induce the carotenogenic genes under the control of copper-inducible GAL system. (B) Carotenoid contents of engineered yeasts in YNBD and YPD media at 72 h. (C) Growth curves of JS-BE5 and JS-BE5-PEST cultivated in YNBD and YPD media. Strain JS-BE5-PEST is derived from JS-BE5 with PEST-Gal80N. (D) Representative HPLC results showing the abundance of carotenoids produced by JS-BE5-PEST. *P* values were obtained from two-tailed unpaired Student’s *t* test. ***P < 0.01. Error bars indicate the SDs of three independent experiments.

Considering that the yeast cells can grow better and accumulate more biomass in the low-cost YPD medium than in YNBD medium, it might facilitate more storage space for lycopene and β-carotene by an increased biomass. Next, we attempted to use the rich YPD medium for carotenoid overproduction. However, we found that both β-carotene and lycopene were poorly produced when using YPD media with rich nutrition (data not shown). We reasoned that the failure of copper-induced GAL expression system in rich YPD media might be caused by the high abundance of Gal80 repressor. To further engineer the GAL system to efficiently function in YPD media, we inserted the PEST sequence at the N terminus of Gal80 into carotenoid-producing strains. As can be seen in Fig. 5A, JS-BE-PEST gave a noticeable orange color on YPD agar plate supplemented with 20 μM copper sulfate, indicating that accelerating the degradation of Gal80 by fusion with the PEST sequence could restore the functionality of copper-inducible GAL system in YPD media. Unlike the conventional method of fusing an N-degron tag (K15) to Gal80, which led to a leaky expression in YNBD medium [42], we found that the PEST-Gal80N system gave a tightly regulated system in response to copper even in YNBD medium (Fig. S1).

As shown in Fig. 5B, JS-BE5-PEST functioned well in both YNBD and YPD media for carotenoid synthesis. It is noteworthy that strain JS-BE5-PEST produced a high level of carotenoids to that of JS-BE5 under YNBD cultivation. In comparison, the β-carotene titer of JS-BE5-PEST in YPD reached 166.79 mg/l, which was ~54.3% improvement over that in YNBD (Fig. 5B). The growth curves based on the OD_600_ measurement showed that the biomass accumulation of JS-BE5-PEST in YPD was much higher than that in YNBD (Fig. 5C), which might be the main reason for an improved β-carotene production. Remarkably, we noticed that YPD medium favored the synthesis of β-carotene over lycopene as there was almost undetectable amount of lycopene (Fig. 5D), indicating that the YPD medium is a good choice for the future β-carotene production. Since the GAL system was recently coupled to the G protein-coupled receptor (GPCR) signaling pathway to achieve the autonomous control of metabolic pathways in a quorum-sensing manner [43], it will be interesting to implement more advanced genetic controls to further simplify the fermentation process.

## Conclusion

In summary, we introduced the NOG pathway to improve the acetyl-CoA supply, reduced the consumption of FPP by inhibiting the ergosterol pathway, introduced the hSapB as a compensatory sink for carotenoid storage, and engineered the GAL expression system to function in YPD media. The combined efforts were capable of promoting the synthesis and accumulation of carotenoids in *S. cerevisiae*. Finally, we obtained an engineered strain of JS-BE5-PEST, which produced 166.79 mg/l β-carotene and less than 0.5 mg/l lycopene in YPD media. Although we did not carry out fed-batch fermentation to further improve the carotenoid titer, it is possible that multidimensional strategies established in this study should be implementable under the large-scale fermentation. In the future, such engineering strategies might be generalizable in other microorganisms for the future industrial development of carotenoid biosynthesis from renewable feedstocks.

## Data Availability

The data involved in the research are included in the manuscript and the Supplementary Materials. All relevant data are available upon reasonable request from the corresponding author.

## References

[1] Takaichi S. Carotenoids in algae: distributions, biosyntheses and functions. Mar Drugs. 2011;9(6):1101–1118. 21747749 10.3390/md9061101 PMC3131562

[2] Borowitzka MA. High-value products from microalgae—their development and commercialisation. J Appl Phycol. 2013;25:743–756.

[3] Gong M, Bassi A. Carotenoids from microalgae: A review of recent developments. Biotechnol Adv. 2016;34(8):1396–1412. 27816618 10.1016/j.biotechadv.2016.10.005

[4] Li J, Zhu D, Niu J, Shen S, Wang G. An economic assessment of astaxanthin production by large scale cultivation of *Haematococcus pluvialis*. Biotechnol Adv. 2011;29(6):568–574. 21497650 10.1016/j.biotechadv.2011.04.001

[5] Mo Q, Song W, Xue Z, Yuan J. Multi-level engineering of *Saccharomyces cerevisiae* for the synthesis and accumulation of retinal. Green Chem. 2022;24:8259–8263.

[6] Ribeiro BD, Barreto DW, Coelho MAZ. Technological aspects of β-carotene production. Food Bioproc Technol. 2011;4:693–701.

[7] Saini RK, Keum YS. Progress in microbial carotenoids production. Indian J Microbiol. 2017;57(1):129–130. 28148991 10.1007/s12088-016-0637-x PMC5243257

[8] Foong LC, Loh CWL, Ng HS, Lan JC. Recent development in the production strategies of microbial carotenoids. World J Microbiol Biotechnol. 2021;37:12. 33392834 10.1007/s11274-020-02967-3

[9] Larroude M, Celinska E, Back A, Thomas S, Nicaud JM, Ledesma-Amaro R. A synthetic biology approach to transform *Yarrowia lipolytica* into a competitive biotechnological producer of β-carotene. Biotechnol Bioeng. 2018;115:464–472. 28986998 10.1002/bit.26473

[10] Jang HJ, Yoon SH, Ryu HK, Kim JH, Wang CL, Kim JY, Oh DK, Kim SW. Retinoid production using metabolically engineered *Escherichia coli* with a two-phase culture system. Microb Cell Fact. 2011;10:59. 21801353 10.1186/1475-2859-10-59 PMC3160355

[11] Yang J, Guo L. Biosynthesis of β-carotene in engineered* E. coli* using the MEP and MVA pathways. Microb Cell Fact. 2014;13:160. 25403509 10.1186/s12934-014-0160-x PMC4239400

[12] Lu Y, Yang Q, Lin Z, Yang X. A modular pathway engineering strategy for the high-level production of β-ionone in* Yarrowia lipolytica*. Microb Cell Fact. 2020;19(1):49. 32103761 10.1186/s12934-020-01309-0 PMC7045511

[13] Zhao X, Shi F, Zhan W. Overexpression of ZWF1 and POS5 improves carotenoid biosynthesis in recombinant* Saccharomyces cerevisiae*. Lett Appl Microbiol. 2015;61(4):354–360. 26179622 10.1111/lam.12463

[14] Yang D, Park SY, Lee SY. Production of rainbow colorants by metabolically engineered *Escherichia coli*. Adv Sci. 2021;8(13): e2100743. 10.1002/advs.202100743 PMC8261500 34032018

[15] Yuan J, Mo Q, Fan C. New set of yeast vectors for shuttle expression in* Escherichia coli*. ACS Omega. 2021;6(10):7175–7180. 33748631 10.1021/acsomega.1c00339 PMC7970545

[16] van den Berg MA, de Jong-Gubbels P, Kortland CJ, van Dijken JP, Pronk JT, Steensma HY. The two acetyl-coenzyme A synthetases of* Saccharomyces cerevisiae* differ with respect to kinetic properties and transcriptional regulation. J Biol Chem. 1996;271(146):28953–28959. 8910545 10.1074/jbc.271.46.28953

[17] Zhou YJ, Buijs NA, Zhu Z, Qin J, Siewers V, Nielsen J. Production of fatty acid-derived oleochemicals and biofuels by synthetic yeast cell factories. Nat Commun. 2016;7:11709. 27222209 10.1038/ncomms11709 PMC4894961

[18] Bogorad IW, Lin TS, Liao JC. Synthetic non-oxidative glycolysis enables complete carbon conservation. Nature. 2013;502(7473):693–697. 24077099 10.1038/nature12575

[19] Meadows AL, Hawkins KM, Tsegaye Y, Antipov E, Kim Y, Raetz L, Dahl RH, Tai A, Mahatdejkul-Meadows T, Xu L, et al. Rewriting yeast central carbon metabolism for industrial isoprenoid production. Nature. 2016;537(7622):694–697. 27654918 10.1038/nature19769

[20] Lin PP, Jaeger AJ, Wu TY, Xu SC, Lee AS, Gao F, Chen PW, Liao JC. Construction and evolution of an *Escherichia coli* strain relying on nonoxidative glycolysis for sugar catabolism. Proc Natl Acad Sci U S A. 2018;115(14):3538–3546. 29555759 10.1073/pnas.1802191115 PMC5889684

[21] Qin N, Li L, Ji X, Pereira R, Chen Y, Yin S, Li C, Wan X, Qiu D, Jiang J, et al. Flux regulation through glycolysis and respiration is balanced by inositol pyrophosphates in yeast. Cell. 2023;186(4):748–763.e715. 36758548 10.1016/j.cell.2023.01.014

[22] Kamineni A, Consiglio AL, MacEwen K, Chen S, Chifamba G, Shaw AJ, Tsakraklides V. Increasing lipid yield in *Yarrowia lipolytica* through phosphoketolase and phosphotransacetylase expression in a phosphofructokinase deletion strain. Biotechnol Biofuels. 2021;14(1):113. 33947437 10.1186/s13068-021-01962-6 PMC8094482

[23] Bu X, Lin JY, Duan CQ, Koffas MAG, Yan GL. Dual regulation of lipid droplet-triacylglycerol metabolism and *ERG9* expression for improved β-carotene production in *Saccharomyces cerevisiae*. Microb Cell Fact. 2022;21(1):3. 34983533 10.1186/s12934-021-01723-y PMC8725481

[24] Ro DK, Paradise EM, Ouellet M, Fisher KJ, Newman KL, Ndungu JM, Ho KA, Eachus RA, Ham TS, Kirby J, et al. Production of the antimalarial drug precursor artemisinic acid in engineered yeast. Nature. 2006;440(7086):940–943. 16612385 10.1038/nature04640

[25] Paddon CJ, Westfall PJ, Pitera DJ, Benjamin K, Fisher K, McPhee D, Leavell MD, Tai A, Main A, Eng D, et al. High-level semi-synthetic production of the potent antimalarial artemisinin. Nature. 2013;496(7446):528–532. 23575629 10.1038/nature12051

[26] Scalcinati G, Knuf C, Partow S, Chen Y, Maury J, Schalk M, Daviet L, Nielsen J, Siewers V. Dynamic control of gene expression in *Saccharomyces cerevisiae* engineered for the production of plant sesquitepene α-santalene in a fed-batch mode. Metab Eng. 2012;14(2):91–103. 22330799 10.1016/j.ymben.2012.01.007

[27] Yuan J, Ching C-B. Dynamic control of *ERG9* expression for improved amorpha-4,11-diene production in *Saccharomyces cerevisiae*. Microb Cell Fact. 2015;14:38. 25889168 10.1186/s12934-015-0220-x PMC4374593

[28] Yang X, Liu J, Zhang J, Shen Y, Qi Q, Bao X, Hou J. Quorum sensing-mediated protein degradation for dynamic metabolic pathway control in *Saccharomyces cerevisiae*. Metab Eng. 2021;64:85–94. 33545357 10.1016/j.ymben.2021.01.010

[29] Deshwal S, Fiedler KU, Langer T. Mitochondrial proteases: Multifaceted regulators of mitochondrial plasticity. Annu Rev Biochem. 2020;89:501–528. 32075415 10.1146/annurev-biochem-062917-012739

[30] Rechsteiner M, Rogers SW. PEST sequences and regulation by proteolysis. Trends Biochem Sci. 1996;21(7):267–271. 8755249

[31] Doshi R, Nguyen T, Chang G. Transporter-mediated biofuel secretion. Proc Natl Acad Sci U S A. 2013;110(19):7642–7647. 23613592 10.1073/pnas.1301358110 PMC3651508

[32] Lee JJ, Chen L, Cao B, Chen WN. Engineering *Rhodosporidium toruloides* with a membrane transporter facilitates production and separation of carotenoids and lipids in a bi-phasic culture. Appl Microbiol Biotechnol. 2016;100(2):869–877. 26526454 10.1007/s00253-015-7102-3

[33] Bu X, Lin JY, Cheng J, Yang D, Duan CQ, Koffas M, Yan GL. Engineering endogenous ABC transporter with improving ATP supply and membrane flexibility enhances the secretion of beta-carotene in *Saccharomyces cerevisiae*. Biotechnol Biofuels. 2020;13:168. 33062054 10.1186/s13068-020-01809-6 PMC7548044

[34] Wu T, Ye L, Zhao D, Li S, Li Q, Zhang B, Bi C. Engineering membrane morphology and manipulating synthesis for increased lycopene accumulation in *Escherichia coli* cell factories. 3 Biotech. 2018;8(6):269. 10.1007/s13205-018-1298-8 PMC5970105 29868307

[35] Ma T, Shi B, Ye Z, Li X, Liu M, Chen Y, Xia J, Nielsen J, Deng Z, Liu T. Lipid engineering combined with systematic metabolic engineering of *Saccharomyces cerevisiae* for high-yield production of lycopene. Metab Eng. 2019;52:134–142. 30471360 10.1016/j.ymben.2018.11.009

[36] Liu Y, Low ZJ, Ma X, Liang H, Sinskey AJ, Stephanopoulos G, Zhou K. Using biopolymer bodies for encapsulation of hydrophobic products in bacterium. Metab Eng. 2020;61:206–214. 32339760 10.1016/j.ymben.2020.04.006

[37] Xu W, Yuan J, Yang S, Ching CB, Liu J. Programming saposin-mediated compensatory metabolic sinks for enhanced ubiquinone production. ACS Synth Biol. 2016;5(12):1404–1411. 27389347 10.1021/acssynbio.6b00148

[38] Son S-H, Kim J-E, Park G, Ko Y-J, Sung BH, Seo J, Oh SS, Lee JY. Metabolite trafficking enables membrane-impermeable-terpene secretion by yeast. Nat Commun. 2022;13(1):2605. 35546160 10.1038/s41467-022-30312-9 PMC9095633

[39] Sun Y, Sun L, Shang F, Yan G. Enhanced production of β-carotene in recombinant *Saccharomyces cerevisiae* by inverse metabolic engineering with supplementation of unsaturated fatty acids. Process Biochem. 2016;51:568–577.

[40] Verwaal R, Wang J, Meijnen JP, Visser H, Sandmann G, van den Berg JA, van Ooyen AJ. High-level production of beta-carotene in *Saccharomyces cerevisiae *by successive transformation with carotenogenic genes from *Xanthophyllomyces dendrorhous*. Appl Environ Microbiol. 2007;73(13):4342–4350. 17496128 10.1128/AEM.02759-06 PMC1932764

[41] Ma Y, Liu N, Greisen P, Li J, Qiao K, Huang S, Stephanopoulos G. Removal of lycopene substrate inhibition enables high carotenoid productivity in *Yarrowia lipolytica*. Nat Commun. 2022;13(1):572. 35102143 10.1038/s41467-022-28277-w PMC8803881

[42] Zhou P, Fang X, Xu N, Yao Z, Xie W, Ye L. Development of a highly efficient copper-inducible *GAL* regulation system (CuIGR) in *Saccharomyces cerevisiae*. ACS Synth Biol. 2021;10(12):3435–3444. 34874147 10.1021/acssynbio.1c00378

[43] Fan C, Yuan J. Reshaping the yeast galactose regulon via GPCR signaling cascade. Cell Rep Methods. 2023;10: 100647. 10.1016/j.crmeth.2023.100647 PMC10753199 37989311

