# Erratum to “Multidimensional Optimization of *Saccharomyces cerevisiae* for Carotenoid Overproduction”

**DOI:** 10.34133/bdr.0061

**Published:** 2024-12-11

**Authors:** Jian Fan, Yang Zhang, Wenhao Li, Zhizhen Li, Danli Zhang, Qiwen Mo, Mingfeng Cao, Jifeng Yuan

**Affiliations:** ^1^ State Key Laboratory of Cellular Stress Biology, School of Life Sciences, Faculty of Medicine and Life Sciences, Xiamen University, Xiamen, Fujian 361102, China.; ^2^ Shenzhen Research Institute of Xiamen University, Shenzhen 518057, China.; ^3^ College of Chemistry and Chemical Engineering, Xiamen University, Xiamen, Fujian 361005, China.; ^4^ Key Laboratory for Synthetic Biotechnology of Xiamen City, Xiamen University, Xiamen, Fujian 361005, China.

In the Research Article “Multidimensional Optimization of *Saccharomyces cerevisiae* for Carotenoid Overproduction”, the authors identified an error in the Results and Discussion section [1]. The amount of β-carotene produced by JS-BE3 was incorrectly listed as 105.94 mg/l. The correct amount is 78.21 mg/l.

The authors apologize for this inadvertent error, which is corrected below and in the original version.

“To limit the EMP pathway for further redirecting the metabolic flux to the NOG pathway, we used the copper-repressible promotor P_CTR1_ to replace the native promoter of phosphoglucose isomerase (encoded by *PGI1*). The resulting strain JS-BE3 (a derivative of JS-BE2 with P_CTR1_-*PGI1*) produced 78.21 mg/l of β-carotene, which represents a nearly 56% improvement compared to JS-BE2. However, there was no significant difference in lycopene contents between strain JS-BE3 and JS-BE2.”
