# Supplementary material for: Multidimensional Optimization of Saccharomyces cerevisiae for Carotenoid Overproduction
Source: Biodes Res. 2024 Jan 10;6:0026. doi: 10.34133/bdr.0026 (PMC10777738; doi:10.34133/bdr.0026)
Supplement: Supplementary 1 — Fig. S1 Tables S1 and S2 [file bdr.0026.f1.docx]

Supplementary Materials

**Multidimensional Optimization of** ***Saccharomyces cerevisiae* for Carotenoid Overproduction**

Jian Fan^1,#^, Yang Zhang^1,4#^, Wenhao Li^1^, Zhizhen Li^1^, Danli Zhang^1^, Qiwen Mo^1^, Qiwen Mo^1^, Mingfeng Cao^2,3^, Jifeng Yuan^1,3,4^*****

^1^ State Key Laboratory of Cellular Stress Biology, School of Life Sciences, Faculty of Medicine and Life Sciences, Xiamen University, Fujian 361102, China

^2^ College of Chemistry and Chemical Engineering, Xiamen University, Fujian 361005, China

^3^ Key Laboratory for Synthetic Biotechnology of Xiamen City, Xiamen University, Fujian 361005, China

^4^ Shenzhen Research Institute of Xiamen University, Shenzhen 518057, China

^#^ These authors contributed equally to the experimental work

* Corresponding author Email: [jfyuan@xmu.edu.cn](mailto:jfyuan@xmu.edu.cn)

**Table S1.** List of primers used in this study

| **Name of primers** | **Sequence** |
| --- | --- |
| R_SUP4 | TTGGTCTCAAAAGAGACATAAAAAACAAAAAAAG |
| F_gRNA.gpp1 | TTGGTCTCAGATGACCGTCAACATCGAATAGAGGTTTTAGAGCTAGAAATAG |
| F_gRNA.gal1 | TTGGTCTCAGATGAGCAACGGCACAAATGAATGGTTTTAGAGCTAGAAATAG |
| F_gRNA.PGI1P | TTGGTCTCAGATGGTAATTCCAGGTTGAGCGCGGTTTTAGAGCTAGAAATAG |
| F_gRNA.ERG9N | TTGGTCTCAGATGCACATATCACACACACACAAGTTTTAGAGCTAGAAATAG |
| F_gRNA.oye2 | TTGGTCTCAGATGTCAATGGAGGAATGACAGCAGTTTTAGAGCTAGAAATAG |
| F_gRNA.Gal80N | TTGGTCTCAGATGGGACTACAACAAGAGATCTTGTTTTAGAGCTAGAAATAG |
| Gpp1_Int_fwd | ATGCCTTTGACCACAAAACCTTTATCTTTGAAAATCAACGAGCGACCTCATGCTATAC |
| Gpp1_Int_rev | TTACCATTTCAACAAGTCATCCTTAGCGTATAAGTAGTCCTTCGAGCGTCCCAAAACC |
| CTR1p_Int_fwd | TATTCTTAGTGGATAACATGCGGCATTTCCTGGCGGCTATGTTATCTAAGCAACTTGGC |
| CTR1p_Int_rev | TGGCCAGTTTGAAGTTAGTGAATGAGTTATTGGACATTTTGAATGTCAAATATAATACAC |
| PEST-ERG9_fwd | GAAAAGACGAAGAGCAGAAGCGGAAAACGTATACACGTGGATCCATGCCAGAATCTACT |
| PEST-ERG9_rev | ATCTCGACCGGATGCAATGCCAATTGTAATAGCTTTCCAGTAGATTCTGGCATGGATCC |
| Oye2_Int_fwd | ATGCCATTTGTTAAGGACTTTAAGCCACAAGCTTTGGGTCGGATTAGAAGCCGCCGAG |
| Oye2_Int_rev | TTAATTTTTGTCCCAACCGAGTTTTAGAGCTTCTTCGTACTTCGAGCGTCCCAAAACC |
| LmXPK_BsaI_fwd | TTGGTCTCATGAAAACAATGAACATTGATTCTACCGA |
| LmXPK_BsaI_rev | TTGGTCTCAATCTTTACTTCAATGGTTGCCATCTC |
| CkPTA_BsaI_fwd | TTGGTCTCAAACCAATGAAGTTGATGGAAAACATC |
| CkPTA_BsaI_rev | TTGGTCTCATCTTAACCTTGAGCTTGGGCTTG |
| Zwf1_BamHI_fwd | CCGGGATCCAAACA ATGAGTGAAGGCCCCGTCAA |
| Zwf1_XhoI_rev | AGAGACTCGAGCTAATTATCCTTCGTATCT |
| hSapB_BamHI_fwd | CCGGGATCCAAACAATGGGCGATGTTTGCCAGGATTG |
| hSapB_XhoI_rev | AGAGACTCGAGTTATTCATCACAGAAGCCAACCAG |

**Table S2.** List of plasmids and strains used in this study.

| **Name** | **Features** | **References** |
| --- | --- | --- |
| **Plasmids** |  |  |
| p415-GPD-Cas9 | Plasmid harboring the Cas9 expression cassette | Lab stock |
| pRS426SNR52 | Plasmid harboring P_SNR52_-T_SUP4_ cassette | Lab stock |
| pRS426-gpp1 | Plasmid harboring P_SNR52_-gRNA.gpp1-T_SUP4_ cassette | This study |
| pRS426-gal1 | Plasmid harboring P_SNR52_-gRNA.gal1-T_SUP4_ cassette | This study |
| pRS426-PGI1p | Plasmid harboring P_SNR52_-gRNA.PGI1P-T_SUP4_ cassette | This study |
| pRS426-ERG9C | Plasmid harboring P_SNR52_-gRNA.ERG9C-T_SUP4_ cassette | This study |
| pRS426-Gal80N | Plasmid harboring P_SNR52_-gRNA.Gal80N-T_SUP4_ cassette | This study |
| pRS425GGA | Plasmid harboring the expression cassette of GAL1/10 | 1 |
| pUC57-hSapB | Plasmid harboring the hSapB gene | 2 |
| pRS425-LmXPK/CkPTA | pRS425GGA derivative with P_GAL10_-*LmXPK*-T_ADH1_; P_GAL1_-*CkPTA*-T_CYC1_ | This study |
| pRS425-ZWF1 | pRS425Gal1 derivative with P_GAL1_-*ZWF1*-T_CYC1_ | This study |
| pRS425-hSapB | pRS425Gal1 derivative with P_GAL1_-*hSapB*-T_CYC1_ | This study |
| **Strain** |  |  |
| *E. coli* Top10 | For cloning purpose | Novagen |
| JS-BE | Strain BY4741 derivative for β-carotene synthesis | 3 |
| JS-BE-PEST | Strain JS-BE derivative with PEST-Gal80N | This study |
| JS-BE2 | Strain JS-BE derivative with ∆gpp1:LmXPK/CkPTA and ∆gal7-10-1:Zwf1 | This study |
| JS-BE3 | Strain JS-BE2 derivative with PGI1 under the control of P_CTR1_ | This study |
| JS-BE4 | Strain JS-BE3 derivative with PEST-ERG9N | This study |
| JS-BE5 | Strain JS-BE4 derivative with ∆oye2:hSapB | This study |
| JS-BE5-PEST | Strain JS-BE5 derivative with PEST-Gal80N | This study |


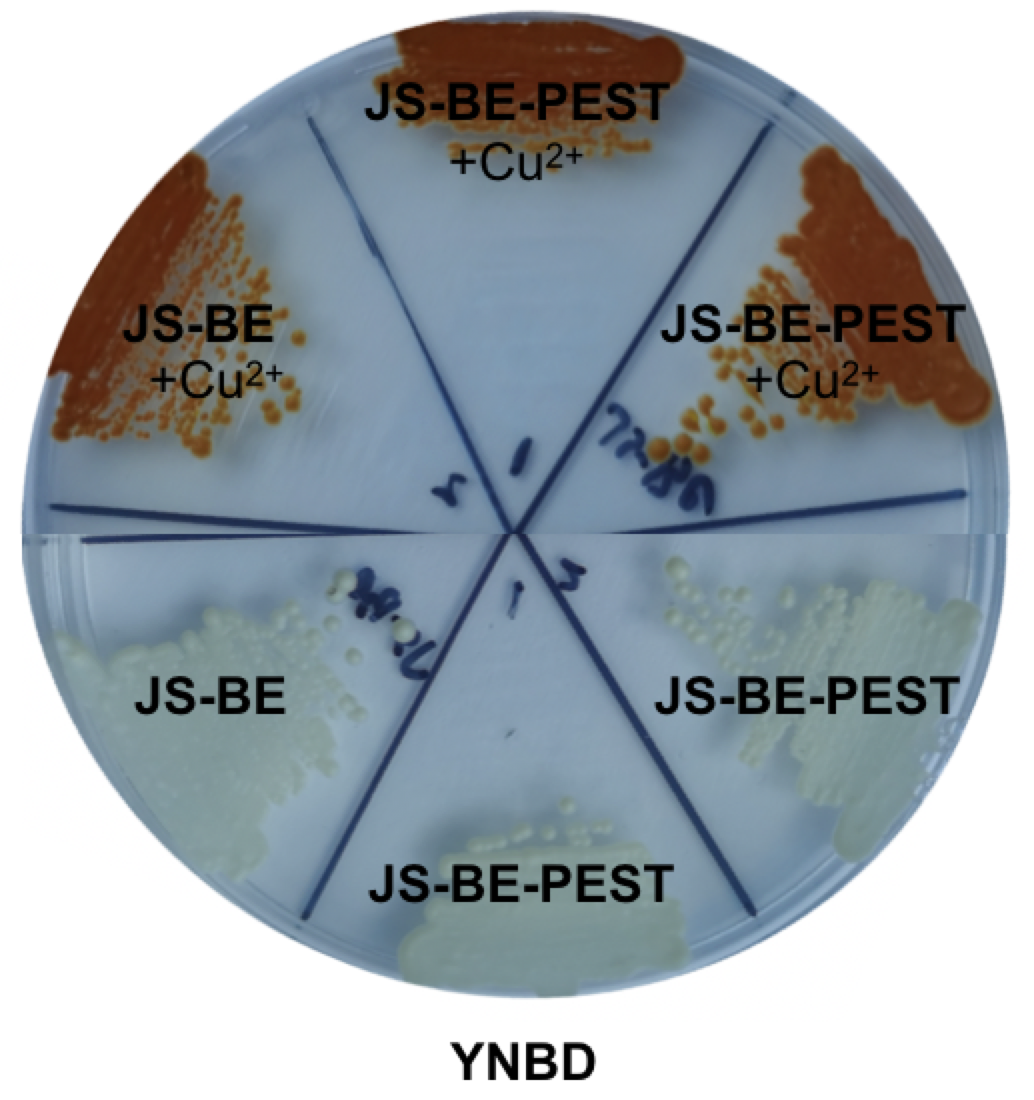


**Figure S1 A PEST fusion to Gal80 for the copper-induced GAL expression in YNBD medium.** 20 µM copper sulfate was used to induce the carotenogenic genes under the control of copper-inducible GAL system. The yeast colonies had the red color appearance on YNBD agar plates supplemented with 20 µM copper sulfate. Images were captured after 3 days.

# References

1. Yuan, J.; Mo, Q.; Fan, C., New Set of Yeast Vectors for Shuttle Expression in Escherichia coli. *ACS Omega* **2021,** *6* (10), 7175-7180.

2. Xu, W.; Yuan, J.; Yang, S.; Ching, C. B.; Liu, J., Programming Saposin-Mediated Compensatory Metabolic Sinks for Enhanced Ubiquinone Production. *ACS Synthetic Biology* **2016,** *5* (12), 1404-1411.

3. Mo, Q.; Song, W.; Xue, Z.; Yuan, J., Multi-level engineering of Saccharomyces cerevisiae for the synthesis and accumulation of retinal. *Green Chemistry* **2022,** *24* (21), 8259-8263.
